# Therapeutic Antithrombotic Agent Use and Bleeding Risk in Retrobulbar Anesthesia for Outpatient Ophthalmic Surgery

**DOI:** 10.7759/cureus.76515

**Published:** 2024-12-28

**Authors:** Kevin Finkel, Edmund T Takata, Aseel Walker, Ya-Huei Li, Ling Lei, Shahad Alassal, David K Emmel

**Affiliations:** 1 Anesthesiology, Integrated Anesthesia Associates, Hartford Hospital, Hartford, USA; 2 Research Administration, Hartford HealthCare System, Hartford, USA; 3 Ophthalmology, Hartford Hospital, Hartford, USA

**Keywords:** antithrombotic therapy, ophthalmic surgery, ophthalmology, retrobulbar anesthesia, retrobulbar hemorrhage

## Abstract

Background and objective

Retrobulbar block is commonly used for providing effective anesthesia and akinesia for ophthalmic surgery. It can, however, lead to sight-threatening retrobulbar hemorrhage in very rare cases. The aim of this study was to evaluate retrobulbar block quality, to determine the prevalence of retrobulbar hemorrhage associated with these blocks, and to assess whether the use of antithrombotic agents in patients undergoing retrobulbar block for ophthalmic surgery was associated with retrobulbar hemorrhage.

Methods

This was a retrospective cohort study conducted on a registry of patients who underwent outpatient ophthalmic surgery at a tertiary care eye center. Adult patients who underwent ophthalmic surgery and received retrobulbar block between October 2014 and December 2021 were retrospectively included to evaluate retrobulbar hemorrhage prevalence and block quality between three groups according to antithrombotic agent use: non-therapeutic antithrombotic use, therapeutic antithrombotic use, and no antithrombotic use.

Results

Of 15,112 records included in final analyses, the median age was 73 with an interquartile range of 67-79 years. White patients totaled 9,813 (64.9%). Patients classified as having mild or major systemic disease before surgery totaled 8,178 (62.9%) and 4,216 (32.4%), respectively. Patients who had at least one comorbidity totaled 13,364 (88.4%). Regarding antithrombotic agent use, 5,856 (38.8%) patients used either one, two, or three medications. Of these, there were 4,183 (27.7%) patients who were therapeutic during the block procedure compared to a total of 10,929 (72.3%) who either did not use antithrombotics or did not use them therapeutically. There were seven (0.1%) retrobulbar hemorrhages among 15,112 patients overall, including two (0.1%) from the 4,183 (27.7%) therapeutic antithrombotic users and five (0.1%) from the 9,256 (61.2%) no antithrombotic users, with no significant differences between groups (p=0.621). Successful blocks totaled 13,364 (95.7%).

Conclusions

Retrobulbar hemorrhage from retrobulbar block in ophthalmic surgery patients is extremely rare, with these results showing no difference in the occurrence of retrobulbar hemorrhage between those using and not using antithrombotic medications. Therefore, it is recommended to continue antithrombotic medication use during ophthalmic procedures as they do not appear to increase the risk of retrobulbar hemorrhage.

## Introduction

The retrobulbar block has been the gold standard anesthesia technique for ophthalmic surgery since its discovery in 1884 as it provides dense anesthesia and akinesia and permits optimal conditions for surgeries to be performed with minimal sedation [1]. Hemorrhagic complications associated with retrobulbar blocks are usually minor, but serious complications such as retrobulbar hemorrhage can occur in very rare cases, resulting in the delay of surgery and even the loss of vision [2-8]. This potential for serious sight-threatening complications has led to the widespread use of theoretically safer techniques such as peribulbar, sub-Tenon's, and local anesthesia. While many ophthalmologists have embraced these newer methods, some still prefer the reliable akinesia, control, and swifter pain relief provided by the retrobulbar block. This is particularly true when the block is performed by an experienced anesthesiologist, often yielding shorter turnover between cases and productivity either equal to or better than topical anesthesia. A review of injection blocks found that while sub-Tenon's block has a preferable safety profile, retrobulbar block promotes superior outcomes compared to peribulbar block when an injection block is indicated [9]. Previous reports have also shown retrobulbar and peribulbar blocks to be similar in quality, according to their ability to provide anesthesia and akinesia [10].

Patients undergoing ophthalmic surgery are often elderly with multiple comorbidities that require the use of antithrombotic agents, including anticoagulant and antiplatelet medications [11]. The American Society of Regional Anesthesia (ASRA) has provided guidance on when these medications should be discontinued before performing interventional pain procedures [12,13]. While there is no such guidance for retrobulbar blocks, data suggest that traditional antithrombotic agents do not affect the incidence of sight-threatening hemorrhages [2-7]. Few studies, however, have evaluated whether antithrombotic agents are associated with the occurrence of major bleeds, such as retrobulbar hemorrhage, due to retrobulbar block. A previous study stated that any differences in risk associated with antithrombotic agents are minimal, but the study only included patients undergoing cataract surgery, and too few complications occurred to draw any meaningful associations [7,14].

Newer-generation anticoagulant (e.g., apixaban {Eliquis®}, dabigatran {Pradaxa®}, and rivaroxaban {Xarelto®}) and antiplatelet medications (e.g., prasugrel {Effient®} and ticagrelor {Brilinta®}) provide additional therapeutic options to patients [11,15-17]. Due to their relatively recent introduction, however, it is unclear whether the concomitant use of these medications increases bleeding risk during a retrobulbar block and, therefore, whether they should be discontinued before surgery. Furthermore, some data suggest that these medications are associated with bleeding following vitreoretinal surgery [18]. However, the discontinuation of antithrombotic therapies can significantly increase the risk of atherothrombotic events [19-21]. Therefore, it is important for studies to quantify the risks, particularly of major bleeding, associated with continuing these therapies in patients undergoing retrobulbar block so clinicians and patients can better determine whether to discontinue antithrombotic medications prior to surgery.

Retrobulbar blocks are routinely performed on patients undergoing ophthalmic surgery at eye surgery centers across the country and at the center included in this study. These patients are also commonly taking one or more antiplatelet or anticoagulant medications. In this retrospective study, clinical records of ophthalmic surgery patients who required a retrobulbar block were reviewed to determine the prevalence of bleeding complications due to retrobulbar block, as well as the rate of block success. This study was undertaken to improve the outcomes of eye blocks at this facility and other similar ophthalmology centers. It was hypothesized that patients on therapeutic antithrombotic use at the time of retrobulbar block would have a similar risk of major bleeding as patients not using antithrombotics at all or using them non-therapeutically. This article was previously presented as a meeting abstract at the 75th Anniversary of the PostGraduate Assembly in Anesthesiology meeting on December 10, 2021.

## Materials and methods

This retrospective cohort study received the Hartford HealthCare Institutional Review Board approval (approval number: E-HHC-2018-021) to be conducted using data from adult patients ≥18 years of age who underwent a retrobulbar block for ophthalmic surgery at the eye surgery center between October 1, 2014, and December 31, 2021. Data of all ophthalmic surgery patients at the eye center were collected from a Research Electronic Data Capture (REDCap) registry licensed by the study institution. Registry data were entered prospectively by anesthesia providers using hardcopy datasheets, which were completed immediately following each retrobulbar block procedure. Data for variables of interest were entered by the anesthesiologist, while data for bleeding complications were entered in the operating room by a certified registered nurse anesthetist who was not involved with the block. Completed datasheets were entered into the prospective registry by a nonclinical research staff member.

Operative globe axial length was measured during the preoperative examination as part of the standard of care. On the day of surgery, all patients were evaluated by an anesthesiologist, and propofol was administered via a peripheral intravenous line for sedation before the eye block. All blocks were performed using the same approach to achieve a retrobulbar block and were administered by either an attending anesthesiologist, a regional anesthesia fellow, an anesthesia resident, or an ophthalmologist.

Retrobulbar blocks were performed via percutaneous inferotemporal injection with or without an additional supraorbital injection, depending on the practitioner administering the block. A local anesthetic mixture of 5-10 mL was administered via a 25 G Atkinson needle. The standard block mixture consisted of equal parts 0.5% bupivacaine with 1:200,000 epinephrine and 2% lidocaine with 1:200,000 epinephrine plus 150 IU of hyaluronidase. Injections were made in divided doses with negative aspiration. Gentle pressure was applied to the ipsilateral eye after the injection to assist in spreading the local anesthesia. Respiration, blood pressure, heart rate, and oxygen saturation were monitored throughout the block procedure.

Registry variables that were collected included age, sex, race, eye axial length, American Society of Anesthesiologists (ASA) physical status, ophthalmic surgery type, and comorbidities. Antiplatelet medications that were recorded included aspirin 81 mg or 325 mg, clopidogrel, ticagrelor, prasugrel, aspirin-dipyridamole, and cilostazol. Anticoagulant mediations that were recorded included dabigatran, rivaroxaban, apixaban, heparin, enoxaparin, and warfarin. The non-therapeutic use of antithrombotic medications was defined as stopping the medication before the time period prior to surgery designated by the ASRA anticoagulation guidelines for regional anesthesia: one day before surgery for aspirin-dipyridamole, heparin, dalteparin, or enoxaparin; two days for cilostazol or dipyridamole; three days for dabigatran, rivaroxaban, edoxaban, apixaban, or acenocoumarol; five days for warfarin, ticagrelor, or dabigatran in a patient with chronic kidney disease; seven days for clopidogrel or aspirin 81 mg or 325 mg; and 10 days for prasugrel [12,13]. The therapeutic use of antithrombotic medications was defined as the continued use of these medications within the time period designated by these ASRA guidelines.

The main factor of interest was the use of therapeutic antithrombotic agents, and several primary comparisons were made according to medication use: three-way comparisons of patient demographics, clinical characteristics, and outcomes among non-therapeutic use, therapeutic use, and no use of antithrombotic medication; two-way comparisons in outcomes between non-therapeutic and therapeutic use of antithrombotic medication and between therapeutic use and no use of antithrombotic medication. The primary outcome was the occurrence of a major bleed, defined as a retrobulbar hemorrhage. The secondary outcome was the rate of successful block, defined as a block resulting in a complete lack of sensation.

Data from this study's retrospective REDCap database were de-identified and exported for statistical analyses. Dichotomous and categorical data were presented as frequency (proportion) and described as mean (standard deviation) or median (interquartile range). Patient demographics, surgical information, comorbidities, and surgical outcomes were analyzed by Kruskal-Wallis or chi-square tests with or without post hoc tests. Additional two-group comparisons on major bleeding (non-therapeutic versus therapeutic antithrombotic users and therapeutic antithrombotic versus no antithrombotic users) were examined using chi-square or Fisher's exact tests. All hypotheses were tested with a two-sided alpha level of 0.05, with all tests performed using SPSS version 29 (IBM Corp., Armonk, NY).

## Results

**Figure 1:**
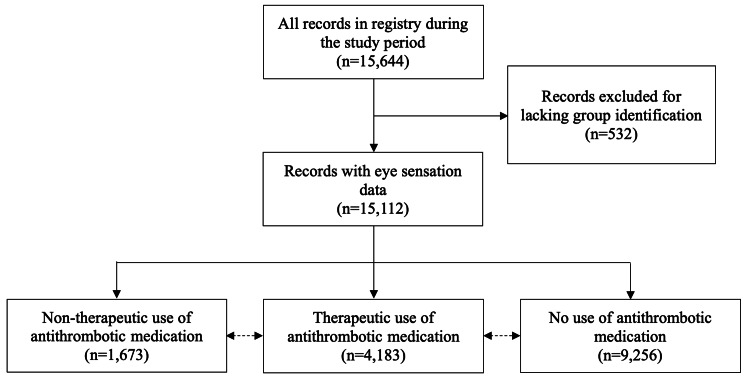
Flowchart of study patient record identification.

As shown in Figure 1, a total of 15,644 patient records were retrieved and assessed for eligibility. Records excluded for lacking information necessary to stratify into groups totaled 532 (3.4%). Of the remaining 15,112 records, seven (0.1%) resulted in a major bleeding event. Records excluded from analyses of block sensation outcome due to missing eye sensation data totaled 1,148 (7.6%), leaving 13,964 (92.4%) records remaining for analyses of the secondary outcome.

Table 1 presents demographics and clinical characteristics for the 15,112 records in total and compared among the three groups. Records in the non-therapeutic antithrombotic use, therapeutic antithrombotic use, and no antithrombotic use groups totaled 1,673 (11.1%), 4,183 (27.7%), and 9,546 (63.2%), respectively. The median age overall was 73 with an interquartile range of 67-79 years. Female patients totaled 7,845 (51.9%); non-Hispanic, 1,102 (72.9%); and White, 9,813 (64.9%). Cataract surgery patients totaled 11,715 (81.9%), and glaucoma totaled 1,374 (9.6%). Patients who reported at least one comorbidity totaled 13,364 (88.4%).

**Table 1:** Population demographics and characteristics stratified and compared among non-therapeutic antithrombotic use, therapeutic antithrombotic use, and no antithrombotic medication use. Data are presented as median (IQR) or frequency (proportion); statistical significance was defined as a p-value of <0.05. IQR = interquartile range presented with 25th-75th percentiles, ASA-I = healthy, ASA-II = mild systemic disease, ASA-III = severe systemic disease, and ASA-IV = severe systemic disease that is a constant threat to life. ^a^Kruskal-Wallis test for non-normally distributed numerical information. ^b^Pearson chi-square test with/without post hoc test for dichotomous or categorical data. ^c^Data of ASA status only available for 13,001 patients due to missing data. COPD, chronic obstructive pulmonary disease; ASA, American Society of Anesthesiologists

Parameter	Total (n=15,112)	Non-therapeutic Antithrombotic Use (n=1,673)	Therapeutic Antithrombotic Use (n=4,183)	No Antithrombotic Use (n=9,256)	Test Statistic	P-Value
Demographics						
Age, years, median (IQR)	73 (67-79)	75 (70-80)	76 (70-82)	72 (65-78)	743.311^a^	<0.001
Sex, n (%)					354.601^b^	<0.001
Female	7,845 (51.9)	814 (48.7)	1,753 (41.9)	5,278 (57.0)		
Male	5,774 (38.2)	723 (43.2)	2,043 (48.8)	3,008 (32.5)		
Unknown/not reported	1,493 (9.9)	136 (8.1)	387 (9.3)	970 (10.5)		
Ethnicity, n (%)					69.242^b^	<0.001
Non-Hispanic	1,1020 (72.9)	1,322 (79.0)	3,126 (74.7)	6,572 (71.0)		
Hispanic	1,169 (7.7)	67 (4.0)	309 (7.4)	793 (8.6)		
Unknown/not reported	2,923 (19.3)	284 (17.0)	748 (17.9)	1,891 (20.4)		
Race, n (%)					95.492^b^	<0.001
White	9,813 (64.9)	1,196 (71.5)	2,833 (67.7)	5,784 (62.5)		
Black	878 (5.8)	77 (4.6)	231 (5.5)	570 (6.2)		
Asian	202 (1.3)	18 (1.1)	24 (0.6)	202 (1.3)		
Other	1,717 (11.4)	134 (8.0)	447 (10.7)	1,136 (12.3)		
Unknown/not reported	2,502 (16.6)	248 (14.8)	648 (15.5)	1,606 (17.4)		
ASA status^c^, n (%)					1,598.044^b^	<0.001
ASA-I	488 (3.8)	25 (1.7)	19 (0.5)	444 (5.6)		
ASA-II	8,178 (62.9)	887 (60.3)	1,535 (41.8)	5,756 (73.2)		
ASA-III	4,216 (32.4)	538 (36.6)	2,054 (56.0)	1,624 (20.7)		
ASA-IV	119 (0.9)	20 (1.4)	60 (1.6)	39 (0.5)		
Comorbidities, n (%)						
Any comorbidity	13,364 (88.4)	1,554 (92.9)	4,073 (97.4)	7,737 (83.6)	571.406^b^	<0.001
Hypertension	9,578 (63.4)	1,241 (74.2)	3,364 (80.4)	4,937 (53.7)	978.978^b^	<0.001
Diabetes mellitus	9,578 (53.4)	415 (24.8)	1,253 (30.0)	1,633 (17.6)	265.475^b^	<0.001
Hypercholesterolemia	5,911 (39.1)	867 (51.8)	2,222 (53.1)	2,822 (30.5)	747.188^b^	<0.001
Peripheral vascular disease	273 (1.8)	25 (1.5)	174 (4.2)	74 (0.8)	184.415^b^	<0.001
Cerebrovascular accident	553 (3.7)	78 (4.7)	363 (8.7)	112 (1.2)	461.126^b^	<0.001
Atrial fibrillation	1,347 (8.9)	275 (16.4)	868 (20.8)	204 (2.2)	1,351.787^b^	<0.001
Myocardial infarction	659 (4.4)	84 (5.0)	465 (11.1)	110 (1.2)	682.843^b^	<0.001
Coronary artery disease	1,567 (10.4)	217 (13.0)	1,024 (24.5)	326 (3.5)	1,375.268^b^	<0.001
Valve disease	346 (2.3)	54 (3.2)	170 (4.1)	122 (1.3)	104.509^b^	<0.001
Congestive heart failure	436 (2.9)	62 (3.7)	225 (5.4)	149 (1.6)	150.600^b^	<0.001
Chronic kidney disease	875 (5.8)	382 (9.1)	382 (9.1)	376 (4.1)	140.754^b^	<0.001
Glaucoma	1,762 (11.7)	520 (12.4)	520 (12.4)	1,041 (11.2)	4.154^b^	0.125
Obstructive sleep apnea	1,084 (7.2)	423 (10.1)	423 (10.1)	518 (5.6)	93.578^b^	<0.001
COPD	1,037 (6.9)	388 (9.3)	388 (9.3)	522 (6.5)	61.161^b^	<0.001
Coagulopathy	40 (0.3)	19 (0.5)	19 (0.5)	15 (0.2)	9.944^b^	0.007
Thrombocytopenia	81 (0.5)	25 (0.6)	25 (0.6)	43 (0.5)	3.008^b^	0.222
Liver dysfunction	134 (0.9)	29 (0.7)	29 (0.7)	95 (1.0)	5.424^b^	0.066
Smoker	378 (2.5)	104 (2.5)	104 (2.5)	243 (2.6)	3.471^b^	0.176
Steroid use	250 (1.7)	73 (1.7)	73 (1.7)	148 (1.6)	0.451^b^	0.798
Surgery type, n (%)					96.219^b^	<0.001
Cataract	11,715 (81.9)	1,384 (84.1)	3,426 (83.3)	6,905 (80.8)		
Glaucoma	1,374 (9.6)	135 (8.2)	388 (9.4)	851 (10.0)		
Plastics	207 (1.4)	40 (2.4)	34 (0.8)	133 (1.6)		
Pterygium	200 (1.4)	7 (0.4)	24 (0.6)	169 (2.0)		
Pterygium-cataract	14 (0.1)	3 (0.2)	4 (0.1)	7 (0.1)		
Retina	20 (0.1)	0 (0.0)	6 (0.1)	14 (0.2)		
Cataract-glaucoma	638 (4.5)	60 (3.6)	197 (4.8)	381 (4.5)		
Cataract-retina	6 (0.0)	0 (0.0)	1 (0.0)	5 (0.1)		
Strabismus	17 (0.1)	2 (0.1)	1 (0.0)	14 (0.2)		
Cornea	117 (0.8)	15 (0.9)	32 (0.8)	70 (0.8)		
Hyphema	1 (0.0)	0 (0.0)	0 (0.0)	1 (0.0)		

The no antithrombotic use group had the lowest median age (72 years) and the lowest rate of having any comorbidity at 7,737 (83.6%) patients, with comorbidities including hypertension, diabetes mellitus, hypercholesterolemia, peripheral vascular disease, chronic kidney disease, cerebrovascular accident, atrial fibrillation, myocardial infarction, coronary artery disease, valve disease, congestive heart failure, obstructive sleep apnea, chronic obstructive pulmonary disease, coagulopathy, and steroid use. Regarding surgery types, compared to the other two groups, fewer no antithrombotic users had cataract surgery, fewer therapeutic antithrombotic users had surgery for plastics, and a higher proportion of no antithrombotic and therapeutic antithrombotic users underwent surgery for pterygium.

Table 2 presents antithrombotic use for each group. Overall, there were 9,256 (61.2%) patients who did not use antithrombotic medications and 5,856 (38.8%) who used antithrombotic medications, with 4,183 (27.7%) who used them therapeutically and 1,673 (11.1%) who used them non-therapeutically. Thus, a total of 10,929 (72.3%) patients either never used antithrombotic medications or discontinued them prior to the therapeutic window.

**Table 2:** Therapeutic anticoagulant and antiplatelet use. Data are presented as frequency (proportion). Therapeutic antithrombotic use is determined based on the American Society of Regional Anesthesia (ASRA) guidelines [12,13].

Number of Antithrombotic Medications	Total (n=15,112)	Therapeutic Antithrombotic Use (n=4,183)	Non-therapeutic Antithrombotic Use (n=1,673)	No Antithrombotic Use (n=9,256)
None	9,256 (61.2)	0 (0.0)	0 (0.0)	9,256 (100.0)
One or more	5,856 (38.8)	4,183 (100.0)	1,673 (100.0)	0 (0.0)
One	5,328 (35.2)	3,692 (88.3)	1,636 (97.8)	0 (0.0)
Two	523 (3.5)	486 (11.6)	37 (2.2)	0 (0.0)
Three	5 (0.0)	5 (0.1)	0 (0.0)	0 (0.0)

Table 3 presents the prevalence of hemorrhage and ecchymosis compared between groups. Major bleeds totaled seven (0.1%), with zero (0.0%) from the non-therapeutic antithrombotic use group, two (0.1%) from the therapeutic antithrombotic use group, and five (0.1%) from the no antithrombotic use group. There was no significant difference in two-group comparisons between non-therapeutic and therapeutic antithrombotic use groups (p=1.000) or between therapeutic and no antithrombotic use groups (p=1.000).

**Table 3:** Hemorrhage and ecchymosis outcomes. Data are presented as frequency (proportion); statistical significance was defined as a p-value of <0.05. ^a^Fisher's exact test for dichotomous or categorical data when 50% of expected counts totaled less than five. ^b^Pearson chi-square test with/without post hoc test for dichotomous or categorical data.

Outcome Variable	Total (n=15,112)	Non-therapeutic Antithrombotic Use (n=1,673)	Therapeutic Antithrombotic Use (n=4,183)	No Antithrombotic Use (n=9,256)	Test Statistic	P-Value
Retrobulbar hemorrhage, n (%)	7 (0.1)	0 (0.0)	2 (0.1)	5 (0.1)	0.021^a^	0.621
Any hemorrhage, n (%)					4.293^b^	0.830
Retrobulbar hemorrhage	7 (0.1)	0 (0.0)	2 (0.1)	5 (0.1)		
Spot hemorrhage	183 (1.2)	18 (1.1)	55 (1.3)	110 (1.2)		
Half-lid ecchymosis	31 (0.2)	4 (0.2)	10 (0.2)	17 (0.2)		
Whole-lid ecchymosis	3 (0.0)	1 (0.1)	0 (0.0)	2 (0.0)		
No bleed reported	14,888 (98.5)	1,650 (98.6)	4,116 (98.4)	9,122 (98.6)		

Table 4 presents eye sensation outcomes for the 13,964 (92.4%) eligible records following the exclusion of 1,148 (7.6%) records of missing eye sensation data. Of the eligible records, blocks resulting in no sensation, partial sensation, and full sensation totaled 13,364 (95.7%), 526 (3.8%), and 74 (0.5%), respectively.

**Table 4:** Quality outcomes among eligible records with eye sensation data. Data are presented as frequency (proportion).

Outcome variables	Total (n=13,964)
Eye sensation, n (%)	
Insensate	13,364 (95.7)
Partial sensation	526 (3.8)
Full sensation	74 (0.5)

## Discussion

This retrospective study examined patients who underwent retrobulbar block anesthesia for ophthalmic procedures and compared the prevalence of major bleeds between three groups of patients: those who were not taking any antithrombotic medications, those who temporarily discontinued taking antithrombotic medications in accordance with the ASRA guidelines resulting in them being outside of the therapeutic window at the time of surgery, and those who continued taking antithrombotic medications within the therapeutic window prior to surgery. The occurrence of a major bleed was extremely low, with only seven cases reported among all patients studied and with no significant difference in the rate of major bleeding occurrence between patients who continued antithrombotic medication therapy, those who discontinued medications, and those who never started medications. This suggests that antithrombotic therapy does not significantly affect bleeding prevalence in eye surgeries needing retrobulbar block.

The prevalence of major bleeding shown in this study is consistent with that from recent reports, which ranges from 0.0019% to 0.44% [14,22-24]. While these rates are low, the potential for sight-threatening injury and even life-threatening systemic complications has forced providers to veer away from the retrobulbar block to theoretically safer procedures with similar anesthesia and akinesia profiles, such as peribulbar and sub-Tenon's block and, in cases where akinesia is not needed, local anesthesia [1,20]. However, there are still cases for which the improved stability, proven reliability, and faster onset of pain relief of the retrobulbar block are preferred, including complex surgical patients, who have undergone repeat ophthalmic procedures and, thus, may have more challenging operations, and obese patients, who often have multiple comorbidities associated with increased risks of complications from sedation or general anesthesia [1]. In addition, retrobulbar block may be useful for patients who are unable to follow verbal commands, such as due to a language barrier. Regarding safety and efficacy, a Cochrane review compared retrobulbar to peribulbar block in 1,438 patients undergoing cataract surgery and found the two to be essentially interchangeable [10], and similar findings have been shown when retrobulbar block is compared to sub-Tenon's block [25]. Thus, even as these newer regional ophthalmic anesthesia techniques continue to gain a share of ophthalmic procedures, the retrobulbar block should see continued use in the foreseeable future for its sustained, proven efficacy and safety.

Antithrombotic medications are often discontinued prior to certain high-risk surgeries due to an increased risk of operative bleeding. In the case of retrobulbar block for ophthalmic surgery, the fear is whether these medications are associated with an increased risk of sight-threatening retrobulbar hemorrhage due to the block. While some have suggested that antithrombotic therapy, particularly dual antiplatelet therapy, is associated with retrobulbar hemorrhage due to retrobulbar block [22], the reported prevalence of these major complications is, as mentioned, very low regardless of antithrombotic medication use and has often been too low for studies to conduct statistical comparisons [14,22,23]. Thus, current evidence suggests that the risks of discontinuing antithrombotic medications likely outweigh the benefits, given that this would increase the risk of life-threatening cardiovascular events while providing little to no protective benefit against the occurrence of retrobulbar hemorrhage [26].

Though there is no standardized method for assessing block quality, the aim of all ocular anesthesia is to achieve akinesia and anesthesia with minimal risk [27]. Retrobulbar block is known to provide at least comparable quality anesthesia relative to sub-Tenon's and peribulbar block, assessed through factors including the ability to provide akinesia and the occurrence of minor bleeding [27]. In this study, retrobulbar block led to adequate anesthesia in 95.7% of patients, which surpassed that of previous findings, suggesting that other factors, such as provider expertise and experience, likely affect block quality [27]. Retrobulbar block has nevertheless been relied on and studied extensively as a widely trusted method of anesthesia for ophthalmic surgery, and these results support its continued ability to provide reliable akinesia and anesthesia [1].

This study has several limitations. First, the institution's record system did not consider that the same individual could have multiple procedures on the same eye, so it did not record that information. Second, since the focus of the registry was to record hemorrhage events of the retrobulbar block, other potentially confounding information might have been omitted. However, the large registry population that was sampled permitted this study to have sufficient statistical power to address whether antithrombotic therapy should be continued or temporarily discontinued before eye surgery.

## Conclusions

This retrospective study highlights that retrobulbar block reliably leads to high-quality akinesia and anesthesia and that retrobulbar hemorrhage is an extremely rare complication that does not appear to be significantly associated with antithrombotic medication use, including for more recently introduced antiplatelet and anticoagulant medications. Therefore, for ophthalmic surgeries requiring retrobulbar block, our data support the decision to continue antithrombotic medication use as they significantly reduce the risk of life-threatening cardiovascular events without appearing to lead to measurably affect the risk of retrobulbar hemorrhage. Despite its extremely rare potential for sight-threatening complications, the retrobulbar block carries advantages particularly for complex cases requiring swift pain relief and akinesia and, therefore, should continue to be relied upon as an effective anesthesia technique alongside newer techniques for ophthalmic surgery.
